# Supplementary material for: Disorder-induced single-mode transmission
Source: Nat Commun. 2017 Mar 6;8:14571. doi: 10.1038/ncomms14571 (PMC5343442; doi:10.1038/ncomms14571)
Supplement: Supplementary Information — Supplementary Figures, Supplementary Notes and Supplementary References. [file ncomms14571-s1.pdf]

Supplementary Note 1: simultaneous activation of multiple single modes

In a disordered optical fiber, it is possible to transmit light on spatially separated transmission channels simultaneously [1]. Here we show that this is possible also exploiting single mode transmission channels. In supplementary Fig. 1 a) we report the experimental setup: in practice the first mode (674 nm laser) is coupled to the input location in the fiber by translating the fiber itself. The second one (532 nm laser) is coupled by keeping the fiber fixed and tilting the input axis. Tilting the laser beam before the objective produces a displacement on the image plane (which is the fiber input tip), in this way it is possible to control the laser at different wavelengths separately. In supplementary Fig. 1 b) we report two single-modes at 532 nm and 674 nm (Dwelling Area (D.A.) of 2.5 µm^2 and 3 µm^2 respectively) coupled separately at two different locations. We also recognized a transmission channel which is serving at the same time both at 532 nm and at 674 nm. This is shown in supplementary Fig. 1 c), d) and e) where the single modes and the two modes superimposed are shown.

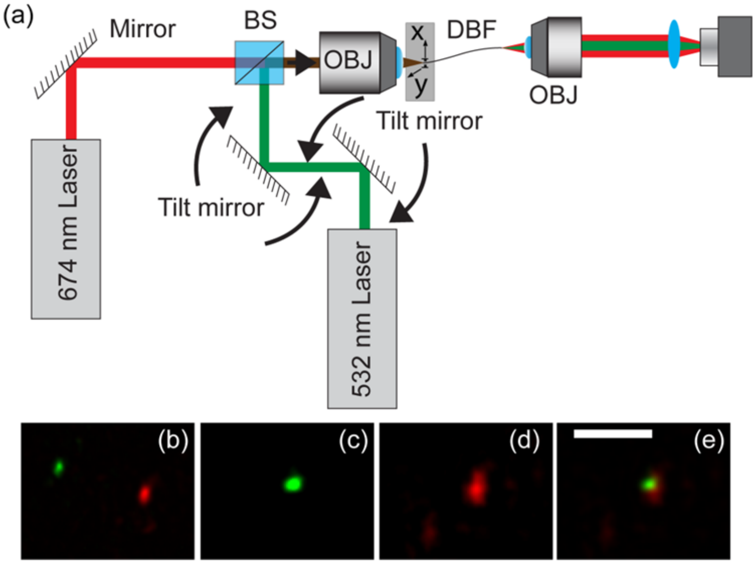

Supplementary Fig. 1 ***Multiple activation of single modes a)*** *Sketch of the experimental setup to couple two different modes to the fiber. light is aligned thanks to the tilt mirrors for the green laser and then coupled and collected thanks to microscopy objectives (OBJ).* ***b)*** *two modes at two different frequencies (674 in red and 532 in green) coupled simultaneously in the fiber. The two modes are single modes (Dwelling areas of 2.5 µm^2 and 3 µm^2 respectively).* ***c)-d)-e)*** *two single modes activated individually and simultaneously (imaging exploiting a dichroic mirror separating the two components and the superimposed digitally).*

Supplementary Note 2: Characterization of the polarization properties

Here we characterize polarization properties of disorder induced single modes. In supplementary Fig. 2(a) we report the experimental setup. In supplementary Fig. 2 (b) we report the map of the transmission efficiency as a function of the input position in the horizontal polarization preserving channel, in supplementary Fig. 2 (c) we repot the vertical polarization preserving channel, while in supplementary Fig. 2 (d) we report the measurement with a vertical input and a non polarized output (analyzer polarized removed). The fact that c) and d) are the same demonstrate that the fiber propagation is essentially polarization preserving. In supplementary Fig. e) we checked the degree of linear polarization of a single mode by scanning the intensity as a function of the analyzer polarizer orientation. The sinusoidal behavior demonstrate the high degree of linear polarization of the output light. A twisting of the fiber by 90 degrees around the propagation axis produced a transmittance map tilted about 90 degrees. The same transmittance map resulted to be resilient to bending up to a bend radius of two centimeters.

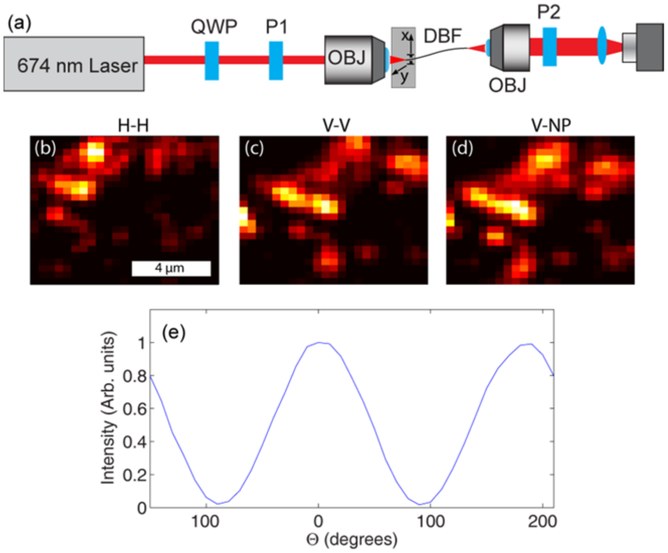

Supplementary Fig. 2 ***Characterization of polarization properties a)*** *Sketch of the experimental setup. Laser light is circularly polarized with a quarter waveplate. The orientation of linear polarization is controlled by the polarized P1 positioned after the waveplate. The transmitted light is measured by exploiting a second polarizer (P2) which is aligned to the input polarization (if the polarization preserving channel is checked) or absent (if all channels are monitored).* ***b)*** *transmittance map for the Horizontal in/ Horizontal out configuration* ***c)*** *transmittance map for the Vertical in/ Vertical out configuration* ***d)*** *transmittance map for the Vertical in/ non polarized configuration. In e) we report the light intensity of a single mode as a function of the orientation of P2 (0 corresponds to the same orientation of the P1).*

Supplementary Note 3: Effects of the input numerical aperture

As shown in supplementary Fig. 2(a), there are large areas of the input providing very low transmittance. In such case rays are either reflected, coupled to lateral leakage or coupled to a guided mode after a relatively long diffusion at the fiber beginning. In the third case, light the rays still arrive (eventually attenuated) at the fiber output, but the initial diffusion makes the output strongly dependent on the input conditions.

The effects of this initial diffusion are unveiled by measurements of the Q-maps of a single mode with varying input numerical aperture (experimental setup shown in Fig 3a)). With a small input numerical aperture, just a single mode is activated. With a larger numerical aperture light which is not coupled to the single mode, produces a background which is masking the single-mode behavior. This effect is due to trajectories which are coupled to guided modes just after a very long diffusion.

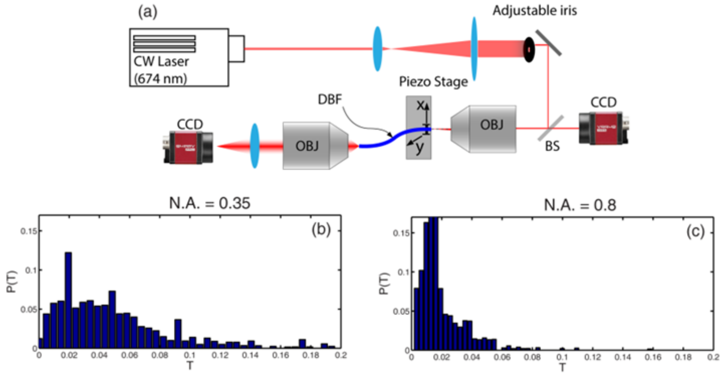

Supplementary Fig. 3 ***Controlling numerical aperture a)*** *experimental setup to measure the response of DBF to varying N.A.. Laser light passes through a beam expander and is then filtered by a adjustable iris. The coupling and collection setup is identical to the one in the main paper. In* ***b)*** *and* ***c)*** *we report the probability distribution of the total transmittance obtained from measurement over a 10x10 μm^2 area (523 locations), for low numerical aperture (panel b), N.A.=0.35), and high numerical aperture (panel c) , N.A.=0.8).*

In the supplementary Fig. 3 (b-c) we report the probability distribution of the transmittance of the fiber for two values of the numerical aperture. The average value of the transmittance decreases from 0.033 to 0.012 when numerical aperture passes from 0.35 to 0.8. the decrease of transmittance confirms that part of the light is not coupled to the target single mode[2], and diffuses until is coupled to a different single mode.

To characterize how the degree of isolation is affected by the residual diffusion, we measured the Q-maps of a single mode (panels in Fig 4 a) as a function of the numerical aperture. The white sharp area in the first panel on the left (N.A. =0.24) indicates a well isolated single mode (D.A.=8 μm^2). On the contrary the blurred boundaries and the lower Q values in the last map on the right (N.A. =0.75, D.A.= 0.75 μm^2) indicate the presence of a speckle background which reduces measured extension of the single-mode. The degree of isolation is quantified by the D.A. of the same mode (Fig. 4 b) which decreases from 8 μm^2 to 0.75 μm^2 confirming that the high numerical aperture introduces superimposes diffusing light to the single mode structure

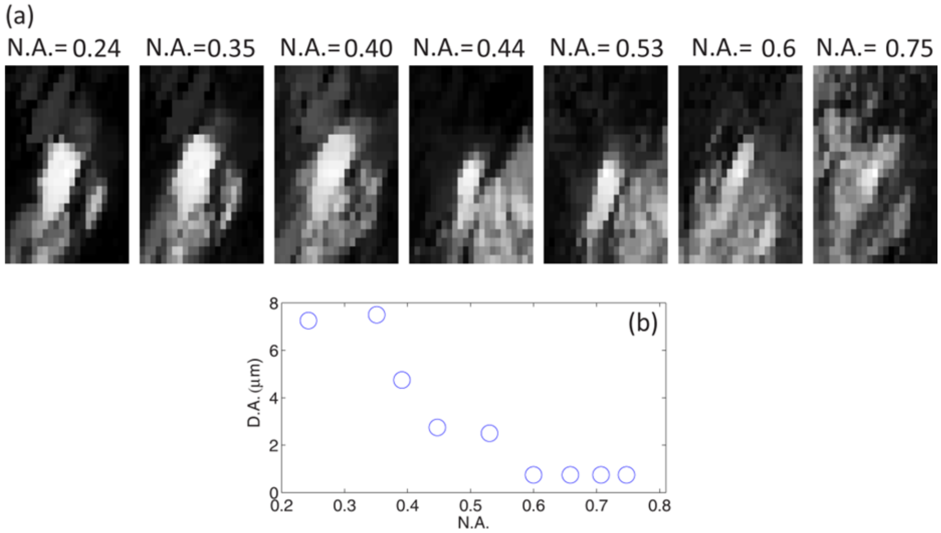

Supplementary Fig. 4 ***Effects of numerical aperture*** **a)** panels: *Q-maps of a single mode feeded with different numerical aperture (indicated on the top of each panel).* ***b)*** *Dwelling area of the same mode as a function of the N.A. .*

We stress that even with very large N.A. the single mode is still there, (input condition may not perturb the mode structure of the fiber), however a large part of the light injected in the fiber is not coupled to it and undergoes diffusion (see for example supplementary Fig. 2 of [3]) before being coupled to a guided mode or lost as lateral leakage. These channels are strongly input dependent and reduce the measured mode's dwelling area.

Supplementary references

[1] S. Karbasi K. W. Koch and A. Mafi; Opt. Expr. 21, 305 (2013).
[2] M. Leonetti, C. Lopez, Opt. Lett. , 36, 2824 (2011).
[3] De Raedt et al. Phys. Rev. Lett. 62, 47 (1989).
